# Ultrabright red AIEgens for two-photon vascular imaging with high resolution and deep penetration†
†Electronic supplementary information (ESI) available: Experimental section, NMR, mass and PL spectrum of TTA, HOMO, LUMO, photophysical properties, cell viability, photostability, and two-photon excited fluorescence spectrum of TTS. See DOI: 10.1039/c7sc04820c


**DOI:** 10.1039/c7sc04820c

**Published:** 2018-01-31

**Authors:** Wei Qin, Pengfei Zhang, Hui Li, Jacky W. Y. Lam, Yuanjing Cai, Ryan T. K. Kwok, Jun Qian, Wei Zheng, Ben Zhong Tang

**Affiliations:** a Department of Chemistry , Hong Kong Branch of Chinese National Engineering Research Centre for Tissue Restoration and Reconstruction , Institute for Advanced Study , Division of Biomedical Engineering , State Key Laboratory of Molecular Neuroscience and Division of Life Science , Hong Kong University of Science and Technology , Clear Water Bay , Kowloon , Hong Kong . Email: tangbenz@ust.hk; b Research Laboratory for Biomedical Optics and Molecular Imaging , Shenzhen Key Laboratory for Molecular Imaging , Institute of Biomedical and Health Engineering , Shenzhen Institutes of Advanced Technology , Chinese Academy of Sciences , Shenzhen , 518055 , China; c NSFC Center for Luminescence from Molecular Aggregate , SCUT-HKUST Joint Research Laboratory , State Key Laboratory of Luminescent Materials and Devices , South China University of Technology , Guangzhou 510640 , China; d State Key Laboratory of Modern Optical Instrumentations , Center for Optical and Electromagnetic Research , JORCEP (Sino-Swedish Joint Research Center of Photonics) Zhejiang University , Hangzhou 310058 , China; e Guangdong Provincial Key Laboratory of Brain Science , Disease and Drug Development , HKUST Shenzhen Research Institute , No. 9 Yuexing 1st RD, South Area Hi-tech Park, Nanshan , Shenzhen 518057 , China

## Abstract

Reported here is a successful strategy for the design of ultrabright red luminogens with aggregation-induced emission (AIE) features, donor–acceptor structures, and intense charge transfer effects. These luminogens show no aggregation caused emission quenching in the solid state and have high quantum efficiency. They can be fabricated into AIE dots by a simple nanoprecipitation procedure. The AIE dots exhibit high brightness, a large Stokes shift, good biocompatibility, satisfactory photostability, and a high two-photon absorption cross section. The AIE dots can be utilized as highly efficient fluorescent probes for *in vivo* deep-tissue imaging by a two-photon technique, which outperforms the one-photon technique under the same experimental conditions, in terms of penetration depth and image contrast. This is the first report of using highly emissive AIE dots for the accurate measurement of capillary diameters in mouse ears. Such a strategy sheds light on the development of efficient solid state red/NIR emitters for biological applications.

## Abstract 2

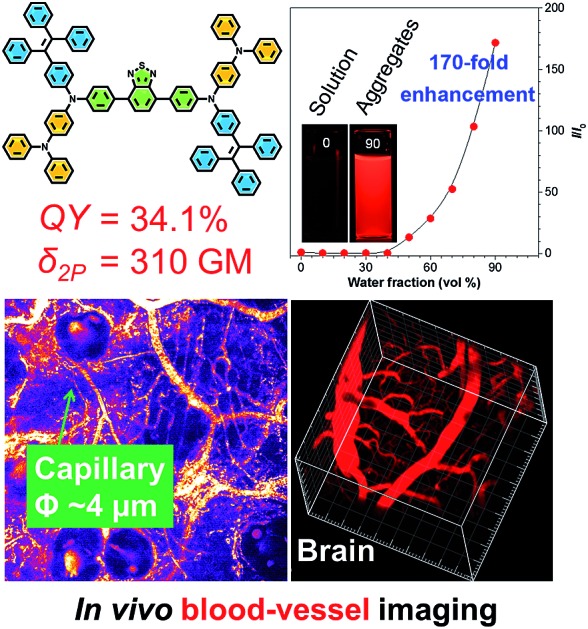
A successful strategy for the design of ultrabright red luminogens with aggregation-induced emission (AIE) features is reported. The AIE dots can be utilized as efficient fluorescent probes for *in vivo* deep-tissue imaging with high penetration depth and high contrast.

## Introduction

Fluorescence imaging is a powerful visualization method that enables real-time following and monitoring of biological processes *in vivo*.^1^ Blood vessels are the primary components of the circulatory system.^2^ The application of fluorescent probes for the visualization of blood vasculature *in vivo* is of great importance as it will shed light on better understanding, medical diagnoses, and therapeutics for common diseases relevant to vascular leakage and obstruction, such as cerebral haemorrhage and cerebral thrombosis.

Two-photon fluorescence microscopy has been widely utilized as an important imaging tool for scientific research due to its high penetration depth with near-infrared (NIR) excitation, high spatial resolution and signal-to-noise ratio, and low tendency for photobleaching.^3^ However, most reports utilize emitters with low two-photon absorption (2PA) cross sections (*δ*_max_ < 50 GM, 1 GM = 1 × 10^–50^ cm^4^ s per photon per molecule)3*c* or with short wavelength emissions.^4^ Due to such reasons, their biological applications are limited to *in vitro*, rather than *in vivo*.3a,4a Thus, fluorescent materials with longer wavelength emissions, high quantum efficiency, and high 2PA cross sections are in urgent demand.

Traditional designs for fluorescent materials with longer wavelength emissions and/or high 2PA cross sections are based on the introduction of nearly planar molecular structures with extended π-conjugation or with strong electron donating (D) and accepting (A) units.^5^ However, these designed chromophores are far from satisfactory as they are prone to aggregation, displaying severe aggregation-caused quenching (ACQ) effects in the aggregated state, which significantly weakens their performance in relevant applications.

**Chart 1:**
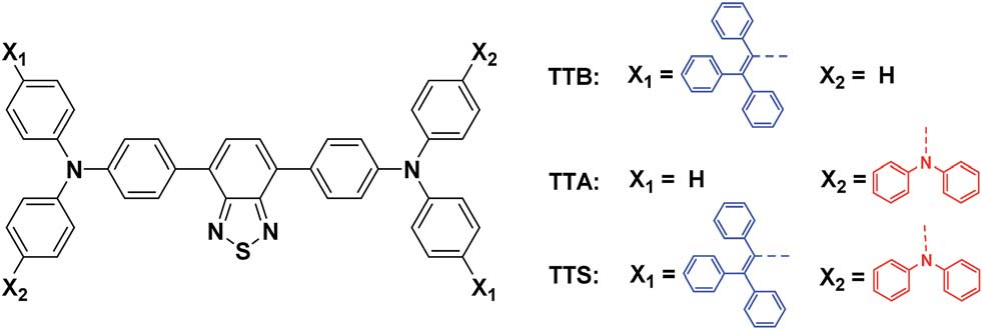
Molecular structure of TTB, TTA, and TTS.

In 2001, we observed the phenomenon of aggregation-induced emission (AIE) in some organic luminophores, which is the exact opposite of the ACQ effect.^6^ Unlike conventional luminophores, these luminophores are non-emissive in dilute solutions, but are induced to emit intensely when aggregated.^7^ Despite the active AIE research in the past decade, only a few red AIE emitters have been reported.^8^ However, most of them show dual twisted intramolecular charge transfer (TICT)^9^ and an AIE effect, with obvious background emission in solution and comparable emission in the aggregated state.8b,8c,^10^ For example, TTB (Chart 1)8*b* was emissive in THF solution and its emission was slightly enhanced when aggregated. Thus, we wonder whether we can solve this problem by suppressing the solution emission; considering TICT and AIE effects are competitive effects, a simple and direct method would be to enhance the TICT effect, which promotes an efficient channel for the excited state to decay non-radiatively.

With this in mind, in this paper we demonstrate a new molecular design strategy through either removing the triphenylvinyl units in TTB and replacing them with a diphenylamino (DPA) functionality, or through simply introducing two DPA units to the TTB structure. Such structural modifications respectively generate TTA and TTS (Chart 1) with weak or no emission in the solution state due to the enhanced TICT effect, although they do show a several hundred-fold stronger emission in the aggregated state because of their AIE effect. Both luminogens exhibit red/infrared emission in the solid state with a high fluorescence quantum yield (*Φ*_F_). The good solubility of TTS enables it to form AIE dots *via* a simple procedure. The AIE dots can be utilized as efficient fluorescent probes for one and two-photon blood vasculature imaging in mouse ears. This is the first report of using highly emissive AIE dots for the accurate measurement of capillary diameters in mouse ears. The AIE dots can be further exploited for real-time imaging of the blood vessels of the brain with deep penetration and high contrast. Following this strategy, many red or NIR emitters with AIE features are expected to be rationally designed.

## Results and discussion

### Synthesis and optical properties of AIE luminogens

TTA and TTS (Chart 1) were prepared according to the synthetic routes shown in Scheme S1 in the ESI.† The chemical structures of the products were confirmed by standard spectroscopic techniques and were of high purity (see Fig. S1–S4 in the ESI†).

In TTA and TTS, the arylamino units serve as electron donors, while the benzothiadiazole core functions as the electron acceptor. The tetraphenylethylene (TPE) in TTS works as the AIE luminogen. A D–A system is constructed to red-shift the absorption and emission to longer wavelengths. The molecular fusion of the D–A system and TPE is expected to generate new AIE-active fluorogens with extended electronic conjugation.8*a* Similar to TTB, TTS shows good solubility in common organic solvents, such as toluene (Tol), 1,4-dioxane (DO), dichloromethane, tetrahydrofuran (THF), and *N*-methylpyrrolidine (NMP), but is insoluble in water. However, TTA is insoluble, or only slightly soluble, in most organic solvents. The low solubility of TTA hampers its purification and further biological applications. Fig. S5† shows the UV spectra of TTA and TTS obtained in different solvents. For comparison, those of TTB are also provided. The absorption maxima of TTA and TTS are slightly red-shifted from that of TTB (Table S1†), possibly due to stronger charge transfer from the additional DPA groups. The ground state electronic transition is affected little by the solvent polarity: all the UV spectra of the studied molecules in different solvents are located at similar wavelengths.

**Fig. 1:**
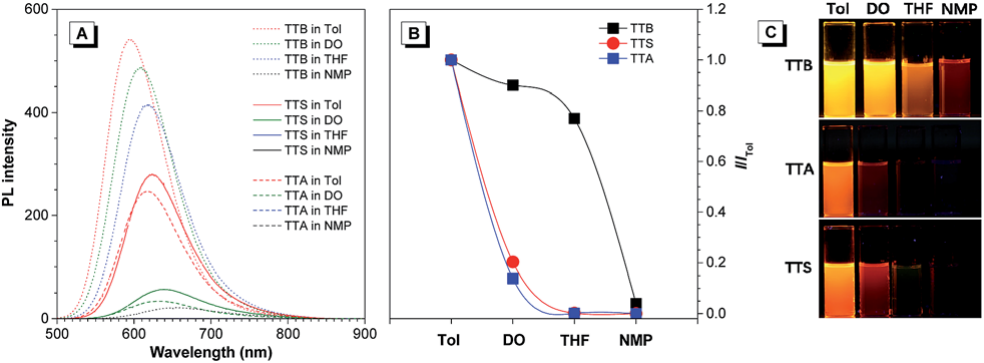
(A) Emission spectra of TTB, TTS, and TTA in solvents with different polarities. Concentration: 10 μM; excitation wavelength: 480 nm. (B) Relative PL intensity (*I*/*I*_Tol_) of TTB, TTA, and TTS in different solvents, where *I*_Tol_ = PL intensity in toluene and *I* = PL intensity in other solvents. (C) Fluorescence photographs of TTB, TTA, and TTS in different solvents taken under 365 nm UV illumination.

The photoluminescence (PL) spectra of TTB, TTA, and TTS obtained in different solvents are shown in Fig. 1. When the solvent polarity is increased from Tol to NMP, the emission of TTB, TTA, and TTS red-shifts with lower intensity. This suggests that they show TICT effects that arise from electron donation from the arylamino units to the benzothiadiazole core.^11^ Compared to that of TTB, the PL of TTA and TTS is more sensitive to the change in solvent polarity. For example, the PL of TTB is still strong enough to be observed by the naked eye in highly polar solvents, such as NMP. In contrast, TTA and TTS already show weak emission in DO and are non-emissive in THF with medium polarity. This clearly shows that the TICT effect becomes stronger by introducing more DPA units into the luminogenic structure. It is presumed that even in THF with medium polarity, TTA and TTS already adopt a twisted conformation. The transition from the locally excited state to the TICT state with increasing solvent polarity causes a red-shift in the PL maximum and emission annihilation.^7^

**Fig. 2:**
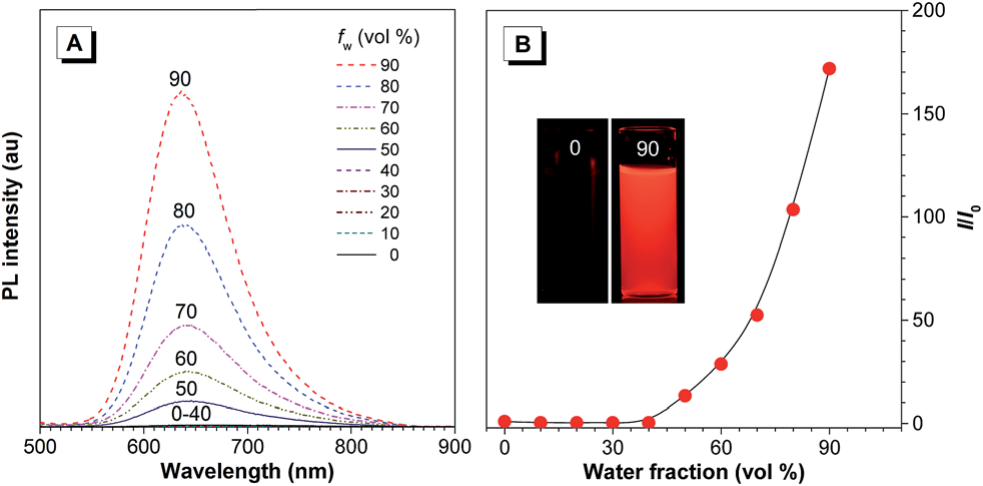
(A) PL spectra of TTS in THF/water mixtures with different water fractions (*f*_w_). (B) Plot of the relative PL intensity (*I*/*I*_0_) *versus* the composition of the THF/water mixture of TTS. *I*_0_ = emission intensity in pure THF solution. Concentration: 10 μM; excitation wavelength: 480 nm. Inset: fluorescence photographs of TTS in THF (*f*_w_ = 0%) and THF/water mixture with *f*_w_ = 90% taken under 365 nm UV illumination.

The PL of TTS is studied in THF/water mixtures with different water fractions (*f*_w_) in order to investigate the effects of solvent polarity and aggregation on its emission process (Fig. 2). The PL spectrum of TTS is basically a flat line parallel to the abscissa in pure THF. The spectral pattern remains unchanged for *f*_w_ up to 40%. Afterwards, the PL intensity increases dramatically. The higher the water fraction, the stronger the emission intensity. The PL maximum moves to ∼636 nm and the emission intensity reaches its maximum value at 90% water content, which is 170-fold higher than that in pure THF solution. TTA shows a similar behavior (Fig. S6†); in pure THF solution, it is non-emissive. The PL intensity remains unchanged for *f*_w_ < 50%, and reaches its maximum value at 90% water content. The PL maximum is located at ∼635 nm. Clearly, TTS and TTA are AIE-active. Quantitative measurements of their *Φ*_F_ in solution and solid states also draw the same conclusion. The *Φ*_F_ of TTS and TTA in pure THF are 0.35% and 0.16%, respectively. In the solid powder state, the *Φ*_F_ becomes 34.1% and 28.0%, respectively (Table S2†). After the covalent integration of TPE, TTS exhibits a 22% enhancement in its solid state emission efficiency compared to TTA. The lifetime of TTS and TTA in solid powders is measured to be 1.86 ns and 6.4 ns, respectively, which rules out the possibility of phosphorescence or delayed fluorescence (Table S2†).

To gain further insight into the photophysical properties of the new molecules at the molecular level, density functional theory calculations on TTS and TTA are performed. The HOMO levels of TTS and TTA were dominated by the orbitals from the benzothiadiazole core and arylamino units. However, the electron clouds of the LUMO levels were mainly located on the core and the adjacent phenyl rings (Fig. S7†). Such electron distributions impart an intrinsic intramolecular charge transfer effect on TTS and TTA. The optimized molecular geometries reveal that TTS and TTA adopt non-planar conformations with different twisting angles between different aromatic rings or double bonds, as they are constructed from the propeller-shaped TPE and twisted arylamino functionalities (Fig. S8–S9 and Table S3–S4†). Such a molecular structure should disfavor close π–π stacking interactions in the aggregated state, which enables it to emit efficiently in the condensed phase.

### Preparation and characterization of AIE dots

**Fig. 3:**
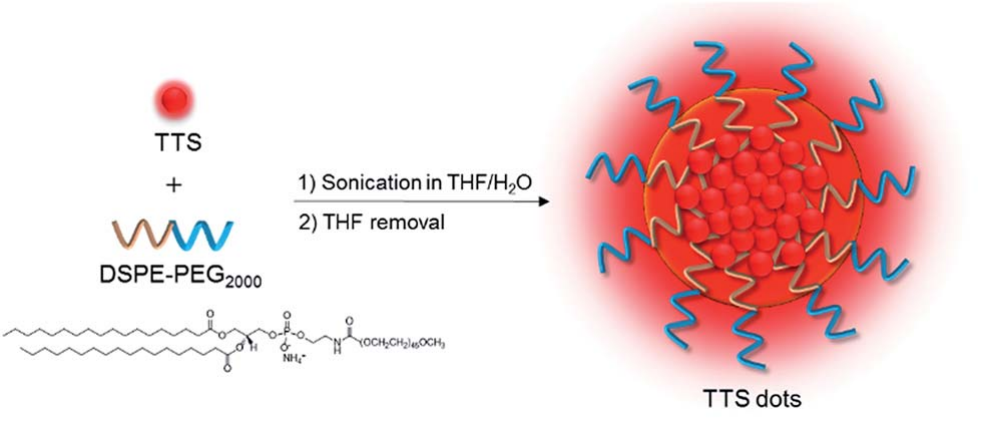
Schematic illustration of AIE dot fabrication.

The red emission, high *Φ*_F_, and good solubility of TTS encourage us to exploit its biological applications. For the easy dispersion of hydrophobic TTS molecules in an aqueous environment, TTS dots were formulated through a simple procedure, using a nanoprecipitation method with amphiphilic and biocompatible 1,2-distearoyl-*sn-glycero*-3-phosphoethanolamine-*N*-[methoxy(polyethyleneglycol)-2000] (DSPE-PEG 2000) as the encapsulation matrix (Fig. 3). The size of the TTS dots was measured by laser light scattering and the hydrodynamic diameter was determined to be ∼120 nm (Fig. S10A†). TTS dots show absorption maxima at 497 nm in aqueous media, which fits well with the commercial laser excitation at 488 nm. The PL spectrum of TTS dots (Fig. S10B†) peaked at 630 nm and extended to the near-infrared region at 820 nm. It is noteworthy that the Stokes shift of the TTS dots is large enough (>130 nm) to solve the problem of emission self-quenching that is commonly observed in conventional organic dyes with small Stokes shifts (usually <25 nm), such as rhodamine, boron dipyrromethane, and cyanine dyes.^12^

**Fig. 4:**
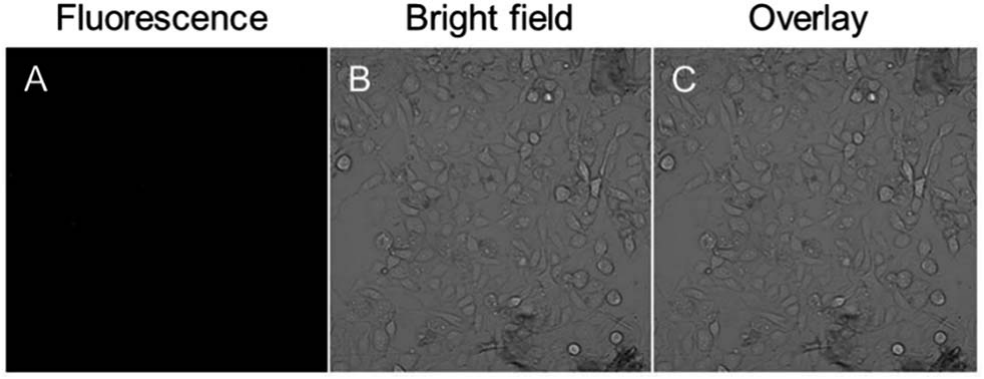
Confocal images of human umbilical vein endothelial cells after incubation with 10 μg mL^–1^ TTS AIE dots for 2 h at 37 °C. (A) Fluorescence image; (B) bright field (C) the merged image of panels (A) and (B).

In order to serve as a vascular contrast agent, one of the essential prerequisites is that the applied dye molecules should show little or no adsorption to the vascular wall. As shown in Fig. 4, no fluorescence signal was detected in the vascular epithelial cells. Generally, in a cultured cell system, the nanoparticles have sufficient time to interact with the cells. However, in a vascular system, the nanoparticles were circulating in the blood vessel. Therefore, they failed to label the cells on the vascular wall. Even for other nanoparticles, which enter the cell through endocytosis, it may not be possible to bind to the blood vessel wall. Our data only showed that the as-prepared nanoparticles showed no non-specific adsorption or endocytosis.

This made them suitable for *in vivo* imaging applications. Fig. S11A† shows the fluorescence loss of the AIE dots and the control rubrene dots under continuous irradiation with a 480 nm Xe lamp. After 12 min irradiation, the AIE dots maintain ∼80% of their initial fluorescence intensity. In contrast, only 25% of the initial value was maintained in the rubrene dots. Clearly, the AIE dots show a better photostability. The cytotoxicity of the AIE dots was evaluated after incubation at different dye concentrations and time intervals. The cell viability remained above 90% when the cells were treated with 0.01, 0.1, and 1 μg mL^–1^ TTS dots within the tested time period (Fig. S11B†). Even at a high dye concentration of 10 μg mL^–1^, the cell viability still remained above 85% after 24 h or 48 h, indicating the low cytotoxicity of the TTS dots. The chemical stability of the TTS dots in aqueous dispersions with different pH was also studied using DLS. As shown in Fig. S12,† the hydrodynamic diameter of the nanoparticles remained at a constant value of ∼100 nm, which demonstrated the high stability of the TTS dots.

### Two-photon excited fluorescence

We next investigated the two-photon absorption spectrum of AIE dots in water at wavelengths from 800 to 1000 nm. The TTS aggregates show strong two-photon absorption (*δ*), with a maximum value of 310 GM and a high quantum efficiency of 38.5% under 900 nm laser excitation (Fig. S13†). The *δ* value of the TTS dots is much higher than that of traditional luminophores, such as fluorescent proteins (commonly <100 GM, and only 39 GM for EGFP),^13^ synthetic GFP-type chromophores (<40 GM),^14^and BODIPY dyes.^15^ Additionally, a 900 nm fs laser is expected to achieve deeper penetration and better focusing capability than commonly used 770–860 nm Ti : Sapphire fs laser. Thus, TTS dots are promising for two-photon deep-tissue *in vivo* bioimaging.

### *In vivo* one and two-photon excited bioimaging

**Fig. 5:**
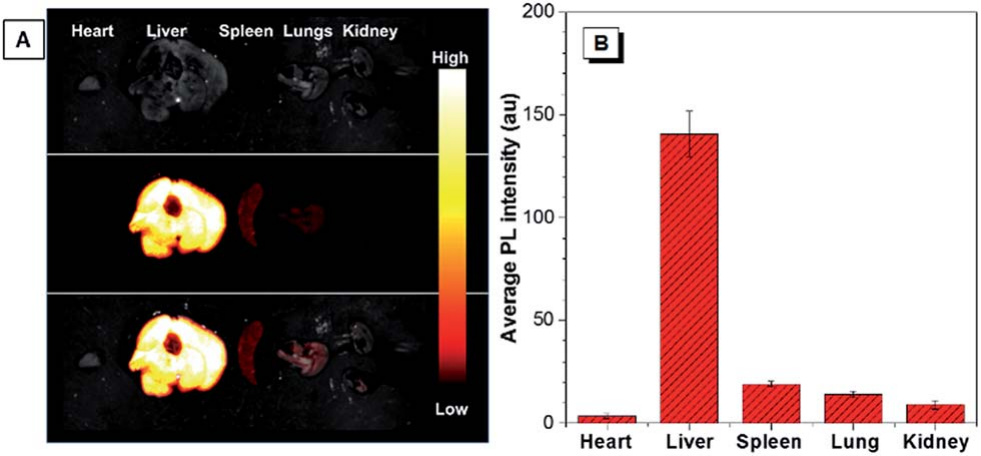
(A) The *ex vivo* bright field (top), fluorescence images (middle), and overlay images (bottom) of the major organs (heart, liver, spleen, lungs, and kidneys) of the sacrificed mice at 3 h post-injection with TTS dots. (B) Average fluorescence intensity distribution for the internal organs of mice sacrificed at 3 h post-injection with TTS dots.

**Fig. 6:**
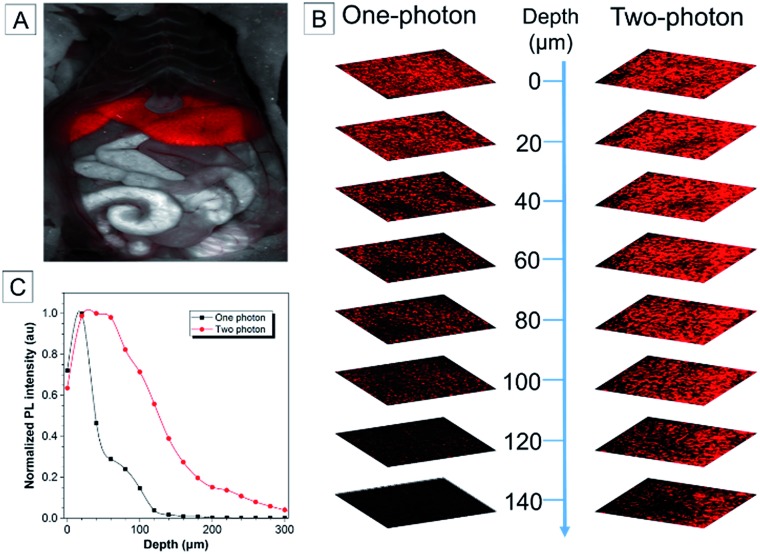
(A) Fluorescence image of the mouse after dissection. (B) *Ex vivo* one-photon confocal fluorescence images and two-photon confocal fluorescence images of the liver of a mouse intravenously injected with TTS dots. (C) Quantitative fluorescence intensity distribution of the liver of mice sacrificed at 3 h post-injection with TTS dots at different penetration depths, from 0 to 300 μm.

To study the biodistribution of AIE-dots in mice, TTS dots were intravenously injected into the tail vein. The major organs of the mouse, including the heart, liver, spleen, lungs and kidneys, were resected at 3 h post-injection and imaged immediately (Fig. 5A). The fluorescence of the TTS dots was mainly observed in the liver, due to the metabolic function of the mouse (Fig. 5B). To obtain further proof, the excised liver was imaged under one (Fig. 6A) and two-photon excitation. Under one-photon excitation (488 nm), strong fluorescence from the TTS dots was observed in the liver at a penetration depth of 40 μm. However, the emission faded quickly at the position of 80 μm and was not detected at 120 μm because of significant absorption/scattering loss (Fig. 6B and C). In contrast, deeper penetration (>200 μm) and mild fluorescence loss were achieved under two-photon excitation (Fig. 6B and C). Clearly, the two-photon fluorescence imaging technique is more advantageous for deep-tissue imaging, with higher penetration depths.

**Fig. 7:**
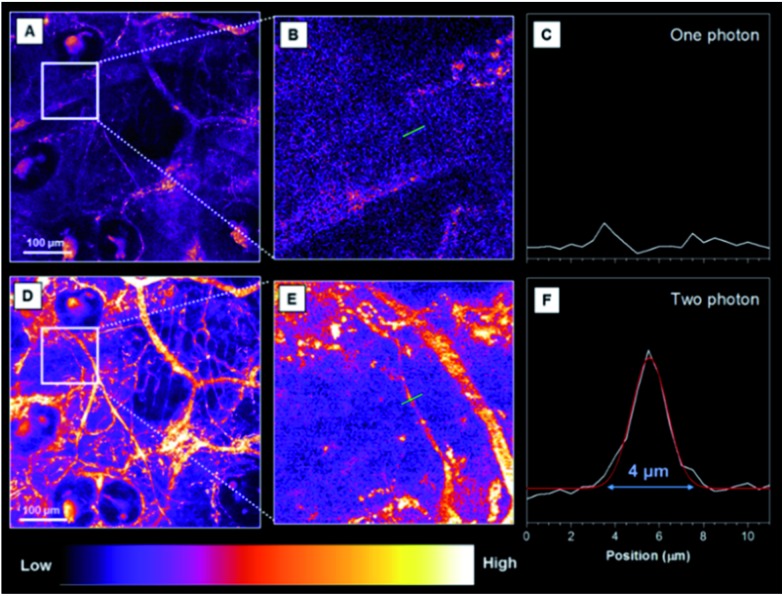
(A) One-photon images (pseudo-color) of the blood vessels in mouse ears after the intravenous injection of TTS dots (z-stage at 110 μm). (B) A zoomed-in image of the selected zone in (A). (C) A cross-sectional intensity profile measured along the green line in (B). (D) Two-photon images (pseudo-color) of the blood vessels in mouse ears after the intravenous injection of TTS dots (z-stage at 110 μm). (E) A zoomed-in image of the selected zone in (D). (F) A cross-sectional intensity profile measured along the green line in (E). Scale bar: 100 μm.

One and two-photon blood vasculature imaging in live mouse ears was then investigated using the AIE dots (Fig. S14†). Red fluorescence was observed in small capillaries at depths of 100–140 μm. However, with the movement of the focus to deeper positions, the one-photon signal decays at a much faster rate than the two-photon one. For further comparison between the two techniques, images at 110 μm are selected and stained with pseudo-color. With one-photon excitation, the signal contrast is low due to the ambiguous signal from the small capillaries and the surrounding tissue (Fig. 7A–C). The image contrast is substantially improved by using the two-photon imaging technique. Careful analysis of Fig. 7D–F enables the quantitative measurement of the diameter of the indicated tiny capillary to be ∼4 μm at a 110 μm depth. To the best of our knowledge, this is the first report using AIE dots for the accurate measurement of capillary diameters in mouse ears. The two-photon imaging technique definitely enables the observation of more fine details of the tiny capillaries with a high signal-to-noise ratio.

**Fig. 8:**
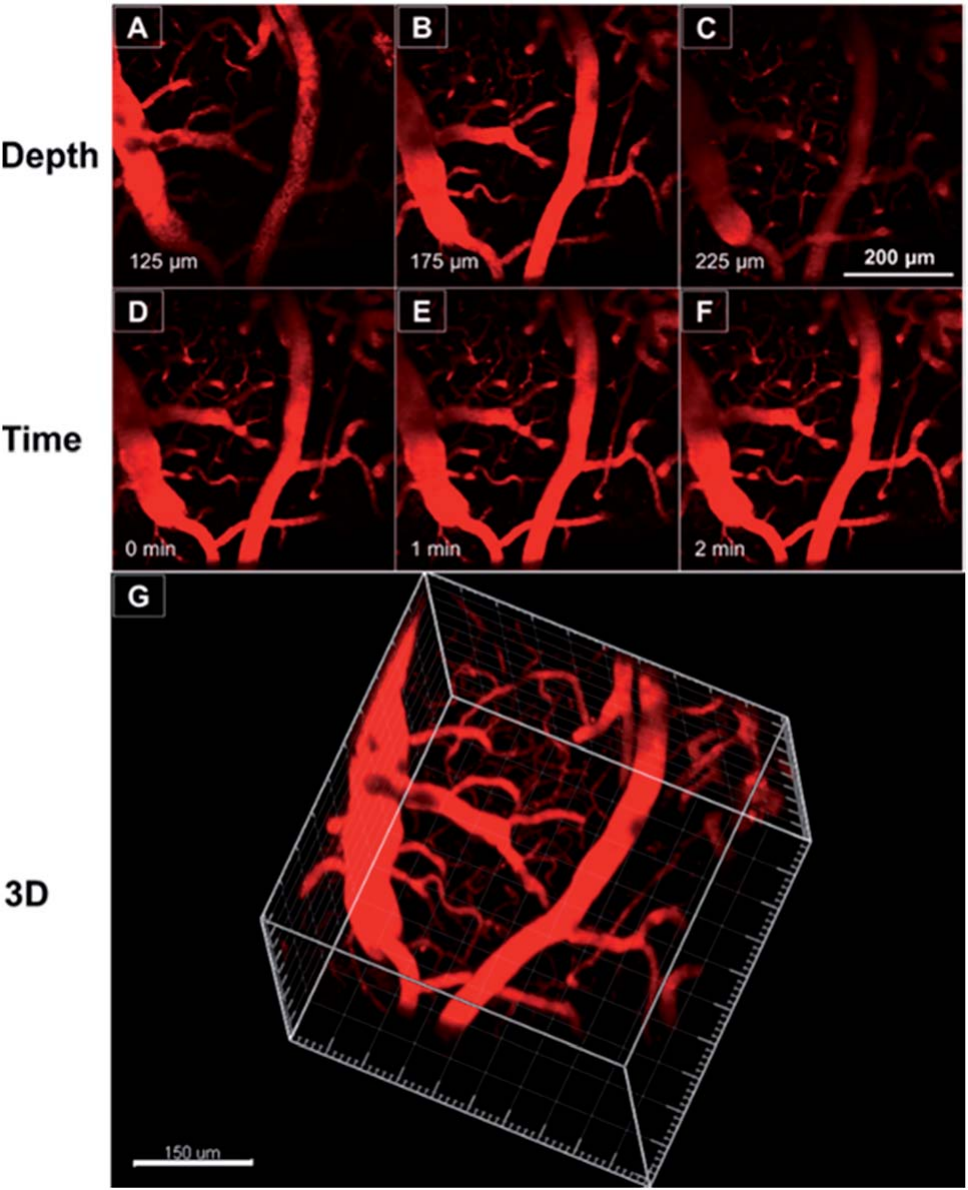
(A–C) Representative two-photon fluorescence images of the blood vessels of a mouse brain, 0.5 h after injection of the TTS dots, at different penetration depths. (D–F) Representative time-lapse images of the blood vessels of a mouse brain, 0.5 h after injection of the TTS dots, at different monitoring time points and at a depth of 200 μm. Scale bar: 200 μm. (G) The constructed 3D image of the blood vessels of the mouse brain, 0.5 h after injection of the TTS dots. Scale bar: 150 μm.

As TTS dots enable deep penetration and high contrast imaging, they were further exploited in the real-time imaging of the blood vessels at a deeper level in mouse brains. Fig. 8A–C show representative vascular images of the mouse brain through a cranial window at penetration depths from 125 μm to 225 μm. The fluorescence signal of the AIE-dots was still detectable at depths of up to 350 μm. Fig. 8D–F are representative high contrast images at different designed time intervals. The dynamic, real-time blood flow process in major blood vessels, and even in small capillaries, could be accurately and vividly displayed by the intense red fluorescence of the AIE dots. A high resolution 3D *in vivo* image was constructed (Fig. 8G), which provided a general and clear spatial picture of the major blood vasculature networks and the details of tiny capillaries. Recently, some red AIE luminogens have been reported for two-photon vascular imaging. However, these molecules have some disadvantages, such as small Stokes shifts (<50 nm)8*c* and relatively low *δ* (<290 GM),8*c* short excitation wavelengths (∼800 nm),^16^ and low quantum efficiency in aqueous media.16b,^17^ Compared to previous studies, the present results are more impressive and are without the obvious shortcomings mentioned above. Thus, AIE dots have successfully realized real-time monitoring of the dynamic blood flow process *in vivo*, with high spatial and temporal resolution.

## Conclusions

In summary, new red-emissive AIE luminogens with insignificant solution emission but efficient aggregate-state PL were designed and synthesized through the enhancement of charge transfer capability and the introduction of stronger D–A interactions. The hydrophobic TTS molecules can be fabricated into AIE dots by a simple procedure. The resulting AIE dots exhibit high brightness, a large Stokes shift, good biocompatibility, satisfactory photostability, and high two-photon absorption cross sections. The AIE dots can be utilized as efficient fluorescent probes for *in vivo* deep-tissue imaging by two-photon excitation, which outperforms one-photon imaging techniques under the experimental conditions. The accurate measurement of capillary diameters in mouse ears is first reported with AIE dots. Such a molecular design strategy opens a new avenue for the development of efficient solid-state red/NIR AIE emitters for wide biological applications, such as following the processes of protein folding and aggregation.

## Conflicts of interest

There are no conflicts to declare.

## Supplementary Material

Supplementary information
