# Supplementary material for: Ultrabright red AIEgens for two-photon vascular imaging with high resolution and deep penetration
Source: Chem Sci. 2018 Jan 31;9(10):2705–10. doi: 10.1039/c7sc04820c (PMC5914133; doi:10.1039/c7sc04820c)
Supplement: Supplementary file 1 [file SC-009-C7SC04820C-s001.pdf]

Electronic Supplementary Material (ESI) for Chemical Science.
This journal is © The Royal Society of Chemistry 2018

Electronic Supplementary Information

Ultrabright Red AIEgens for Two-Photon Vascular Imaging with High Resolution and Deep Penetration

Wei Qin [a#], Pengfei Zhang [a#], Hui Li [b#], Jacky W. Y. Lam [a], Yuanjing Cai [c], Ryan T. K. Kwok [a], Jun Qian [d], Wei Zheng [b] and Ben Zhong Tang [ace*]

[a.] *Department of Chemistry, Hong Kong Branch of Chinese National Engineering Research Centre for Tissue Restoration and Reconstruction, Institute for Advanced Study, Institute of Molecular Functional Materials, Division of Biomedical Engineering, State Key Laboratory of Molecular Neuroscience, Division of Life Science, Hong Kong University of Science and Technology, Clear Water Bay, Kowloon, Hong Kong. E-mail: tangbenz@ust.hk*

[b.] *Research Laboratory for Biomedical Optics and Molecular Imaging, Shenzhen Key Laboratory for Molecular Imaging, Institute of Biomedical and Health Engineering, Shenzhen Institutes of Advanced Technology, Chinese Academy of Sciences, Shenzhen, 518055, China.*

[c.] *NSFC Center for Luminescence from Molecular Aggregates, SCUT-HKUST Joint Research Laboratory, State Key Laboratory of Luminescent Materials and Devices, South China University of Technology, Guangzhou 510640, China.*

[d.] *State Key Laboratory of Modern Optical Instrumentations, Center for Optical and Electromagnetic Research; JORCEP (Sino-Swedish Joint Research Center of photonics) Zhejiang University, Hangzhou 310058, China.*

[e.] *Guangdong Provincial Key Laboratory of Brain Science, Disease and Drug Development, HKUST Shenzhen Research Institute, No. 9 Yuexing 1st RD, South Area Hi-tech Park, Nanshan, Shenzhen 518057 China.*

[#] *W. Qin, P. Zhang and H. Li contributed equally to this work.*

Experimental Section

Materials and Instrumentation

Chemicals for synthesis were purchased from Sigma-Aldrich or J&K used as received without any further purification. 4-bromotriphenylamine (**3**),[1] TPE derivative (**6**),[2] 4,7-bis(4-bromophenyl)-2,1,3-benzothiadiazole (**10**),[3] were prepared according to the reported literatures. THF was distilled from sodium benzophenone ketyl under dry nitrogen before use. 1,2-Distearoyl-*sn*-glycero-3-phosphoethanolamine-*N*-[methoxy(polyethylene glycol)-2000] (DSPE-PEG_{2000}) was a commercial product of Avanti Polar Lipids, Inc. (USA). ^{1}H- and ^{13}C-NMR spectra were carried out on a Bruker AV 400 spectrometer. High resolution mass spectra (HRMS) were recorded on a GCT premier CAB048 mass spectrometer operating in MALDI-TOF mode. Ultraviolet–visible (UV-vis) absorption spectra were taken on a PerkinElmer Lambda 25 UV-Vis absorption spectrophotometer. Photoluminescence spectra were recorded on a PerkinElmer LS 55 fluorescence spectrometer. The solution fluorescence quantum

yield was determined by Rhodamine 6G in ethanol as a standard (Φ_F = 94%). The absorbance of the solutions was maintained below 0.1 to avoid the internal filter effect. The emission efficiency of solid powders of TTS and TTA was measured on a calibrated integrating sphere with 480 nm excitation. The average particle size and size distribution were determined by laser light scattering with a particle size analyzer (90 Plus, Brookhaven Instruments Co. USA) at a fixed angle of 90° at 24°C.

Preparation of TTA and TTS nanoaggregates

Stock THF solutions of TTA and TTS were prepared with a concentration of 10^{-4} M. Aliquots of the stock solution were transferred to 10 mL volumetric flasks. After appropriate amounts of THF were added, water was dropwise added under stirring to prepare solutions with a concentration of 10^{-5} M with different water fractions (0–90 vol%). The PL measurements of the resulting solutions were immediately carried out.

Measurements of two-photon absorption cross-section of TTS

The two-photon absorption cross section measurements were carried out on an Avesta TiF-100M femtosecond Ti:sapphire oscillator as the excitation source. The output laser pulses had a pulse duration of about 80 fs with a repetition rate of 84.5 MHz in the wavelength range from 800 to 1000 nm. The laser beam was focused onto TTS dots in a cuvette with a path length of 1.0 cm. The emission was collected at an angle of 90° to the incoming excitation beam by a pair of lenses and an optical fiber connected to a monochromater (Acton, Spectra Pro 2300i) charge-coupled device (Princeton Instruments, Pixis 100B) system. A short-pass filter with a cutoff wavelength at 750 nm was placed before the spectrometer to remove scattering from the pump beam. Rhodamine B with concentration of 1uM in methanol was utilized as reference.

Preparation and characterization of AIE-dots

AIE dots were formulated through the method of nanoprecipitation procedure. Briefly, 0.25 mL of TTS solutions in THF (1 mg/mL) and a certain amount of DSPE-mPEG2000 solutions in THF (1 mg/mL) were mixed in an eppendorf tube (1.5 mL). After dilution to 1 mL with THF, the mixture was quickly added to 5 mL MilliQ water under the ultrasonication for several minutes to form a homogeneous red solution using a microtip probe sonicator (XL2000, Misonix Incorporated, NY). The mixture was then stirred at room temperature overnight to remove THF and filtered by a syringe filter (0.22 μm) to yield the TTS dots. The TTS dots solutions were concentrated to 1mg/mL using Millipore Amicon Ultra (100 kDa) centrifugal filter tubes.

Cytotoxicity of TTS

Cell Counting Kit-8 based cell viability assay was carried out to evaluate the metabolic activity of cells. A549 cells were seeded in 96-well plates (Costar, IL) at a density of 6 × 10^{-4} cells/mL. After 24 h incubation, the old medium was replaced by TTS suspension with different concentrations ranging from 0.01 to 10 μg/mL, and the cells were then incubated for 24 and 48 h. Then, the wells were washed with 1×PBS buffer and 10 μL CCK-8 Reagent was added into each well. After 3 h incubation at 37°C, the absorbance

of CCK-8 at 450 nm was monitored by the microplate reader. Cell viability was expressed by the ratio of absorbance of the cells incubated with nanoparticles solution to that of the cells incubated with culture medium only.

Animals

Female BALB/c nude mice (weighed 20-22 g and 5-6 weeks old) were purchased from Vital River Laboratory Animal Technology Co. Ltd (Beijing, China) and all animals received care in compliance with the guidelines outlined in the guide for the Care and Use of Laboratory Animals. The procedures were approved by Animal Care and Use Committee of Shenzhen Institutes of Advanced Technology, Chinese Academy of Sciences.

***In vivo* imaging of ear blood vessels**

100 μL PBS solution of TTS dots (0.1 mg/mL) were intravenously injected into the nude mice. The mice in the control group were intravenously injected with 100 μL PBS solution. The mice were anesthetized and placed on a Petri dish with one ear attached to the coverslip, and put under the upright two-photon fluorescence scanning microscope (Nikon, A1 MP+) for imaging. The 488 nm CW laser beam and the 900 nm fs laser beam was focused by the water-immersed objective lens (25×, NA = 1.10) onto the earlobe immersed in water, respectively. The one-photon or two-photon fluorescence signals of TTS dots (from the mouse ear) passed through a 590 nm long pass filter, and collected by a photomultiplier tube (PMT) directly or via nondescanned detection (NDD) mode, respectively.

Biodistribution of TTS AIE-dots in mice

Mice were intravenously injected with suspensions of TTS AIE-dots (0.1 mg/mL in phosphate buffered saline, 100 μL). The mice were sacrificed after 3 h injection. Three mice were used at each time after injection. Their major organs (heart, lung, liver, spleen, and kidney) were taken out and imaged by utilizing a Maestro in vivo optical imaging system (CRI, Inc. Woburn, MA). The organs of mice were imaged with 595 nm excitation and 620-700 nm emission. The fluorescence images were recorded with a constant exposure time of 2000 ms for all the samples. The average fluorescence intensities were calculated by the following formula: average signals (counts/s/pixel) = total counts/exposure time (s)/area (pixel). To compare the microcosmic one-photon fluorescence (1 PL) and two-photon fluorescence (2 PL) signals quantitatively from TTS dots, the 1 PL and 2 PL of the liver tissue were performed by a CW laser at 488 nm and a femtosecond laser at 900 nm, respectively. A water-immersion objective lens (25×, NA = 1.10) was used to focus the laser beam onto the samples, and an external detector was used to collect emission signals.

***In vivo* imaging of brain blood vessels**

A cranial window microsurgery on the mouse brain was carried out. Briefly, mice were anesthetized and a small piece of skull was opened up with a dental drill. The surgery was operated under sterile conditions to avoid infections and damages to the dura mater,

as well as to ensure that the mice could live well in the whole imaging process. Then, the mice were injected intravenously with PBS solution of TTS dots (100 μL), and placed under the above mentioned two-photon fluorescence scanning microscope after anesthetized. The two-photon fluorescence signals of TTS dots (from the mouse brain) passed through a 590 nm long pass filter, and collected by a photomultiplier tube (PMT) via non-descanned detection (NDD) mode.

Synthesis

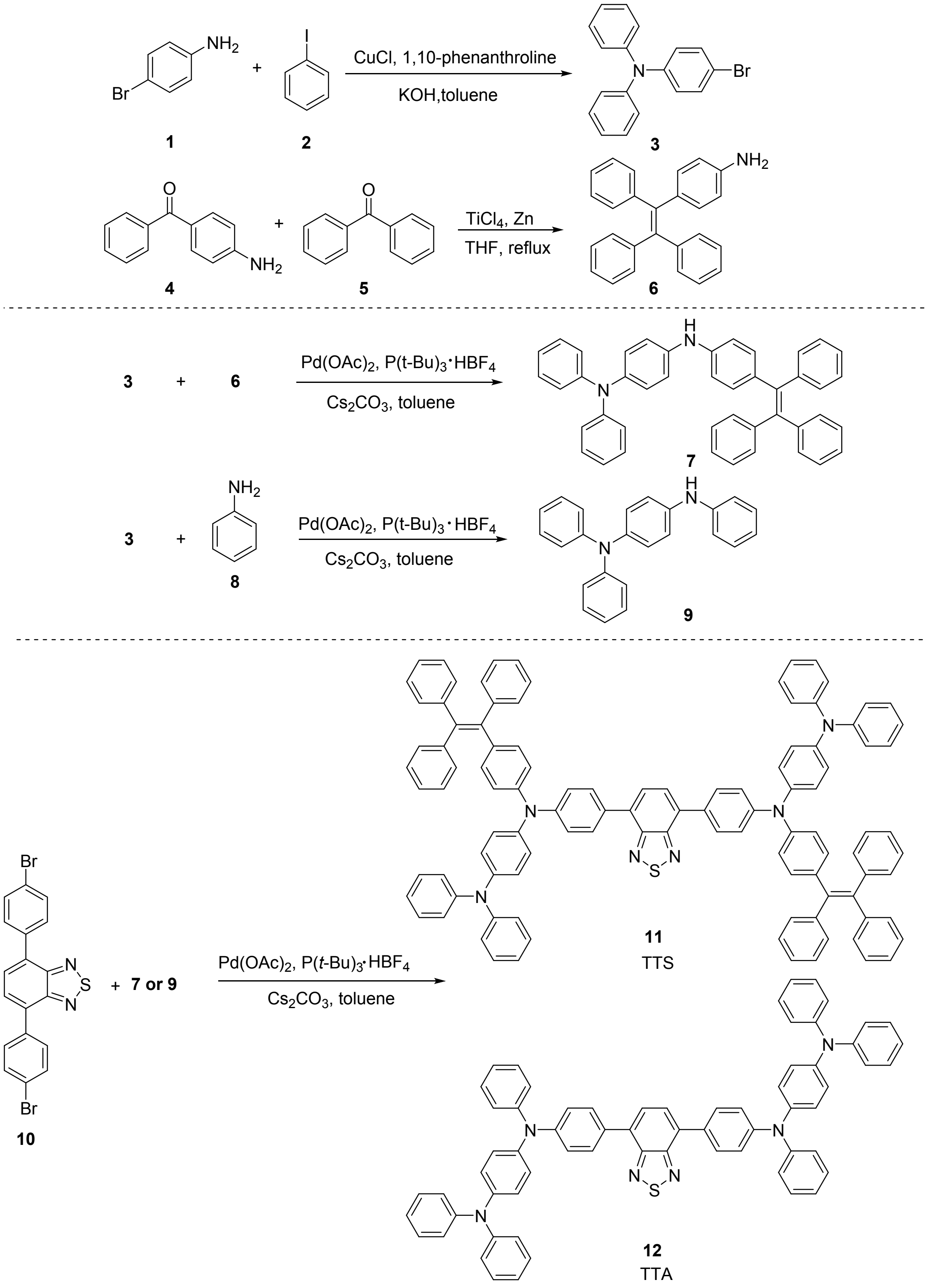

Scheme S1. Synthetic routes to TTS and TTA.

Preparation of N^1,N^1-diphenyl-N^4-(4-(1,2,2-triphenylvinyl)phenyl)benzene-1,4-

diamine (**7**): 4-bromotriphenylamine **3**, (810 mg, 2.5 mmol), 4-(1,2,2-triphenylvinyl)aniline **6**, (868 mg, 2.5 mmol), $Pd(OAc)_2$ (22.4 mg, 0.1 mmol), P(*t*-Bu)$_3$·HBF_4 (58 mg, 0.2 mmol), Cs_2CO_3 (1.63 g, 5.0 mmol) and dry toluene (40 mL) was heated at 45 °C for 1 h under nitrogen. The reaction mixture were then heated at 90 °C for 36 h. Afterwards, the mixture was cooled to room temperature. After filtration, water (60 mL) and dichloromethane (150 mL) were added. The organic layer was separated, washed with brine, dried over anhydrous $MgSO_4$ and evaporated to dryness under reduced pressure. The crude product was purified by silica gel column chromatography on using hexane/dichloromethane as eluent to afford **7** as pale yellow powders in 60.2% yield (888 mg). ^{1}H NMR (400 MHz, THF-d_8), δ (TMS, ppm): 7.35 (s, 1H), 7.21–7.16 (m, 4H). 7.13–6.95 (m, 23H), 6.91 (t, J = 7.6 Hz, 2H), 6.82 (dd, J = 8.8 Hz, J = 2.0 Hz, 4H). ^{13}C NMR (100 MHz, THF-d_8), δ (TMS, ppm): 149.20, 145.36, 145.20, 145.15, 143.69, 142.01, 141.41, 140.55, 140.38, 135.70, 133.12, 132.31, 132.22, 132.18, 129.76, 128.42, 128.29, 127.50, 127.02, 126.90, 126.82, 123.75, 122.54, 119.66, 119.59, 116.15, 116.10; HRMS (MALDI-TOF, *m*/*z*): $[M]^+$ calcd. for $C_{44}H_{34}N_2$, 590.2722; found, 590.2701.

Preparation of N^1,N^1,N^4*-triphenylbenzene-1,4-diamine* (**9**): The compound was prepared from 4-bromotriphenylamine **3**, (810 mg, 2.5 mmol), aniline **8**, (279 mg, 3 mmol), $Pd(OAc)_2$ (22.4 mg, 0.1 mmol), P(*t*-Bu)$_3$·HBF_4 (58 mg, 0.2 mmol) and Cs_2CO_3 (1.63 g, 5.0 mmol), following the same procedure described above. Yield: 61.1% (513 mg). ^{1}H NMR (400 MHz, DMSO-d_6), δ (TMS, ppm): 8.14 (s, 1H), 7.24–7.17 (m, 6H). 7.05–7.02 (m, 4H), 6.95–6.90 (m, 8H), 6.77 (t, J = 7.6 Hz, 1H). ^{13}C NMR (100 MHz, DMSO-d_6), δ (TMS, ppm): 147.61, 143.53, 139.98, 139.05, 129.23, 129.12, 127.00, 122.20, 121.72, 119.43, 118.09, 116.47. HRMS (MALDI-TOF, *m*/*z*): $[M]^+$ calcd. for $C_{24}H_{20}N_2$, 336.1626; found, 336.1627.

Preparation of TTS: A mixture of compound **10**, (134 mg, 0.3 mmol), N^1,N^1*-diphenyl-*N^4*-(4-(1,2,2-triphenylvinyl)phenyl)benzene-1,4-diamine* **7**, (413 mg, 0.7 mmol), $Pd(OAc)_2$ (27 mg, 0.12 mmol), P(*t*-Bu)$_3$·HBF_4 (104 mg, 0.36 mmol), Cs_2CO_3 (975 mg, 3.0 mmol) and dry toluene (40 mL) was heated at 45 °C for 1 h under nitrogen. The reaction mixture was then heated at 100 °C for 36 h. Afterwards, the mixture was cooled to room temperature. After filtration, water (30 mL) and dichloromethane (200 mL) were added. The organic layer was separated, washed with water three times, dried over anhydrous Na_2SO_4 and evaporated to dryness under reduced pressure. The crude product was purified by silica gel column chromatography using hexane/dichloromethane as eluent, affording orange red solid in 61.6% yield (271 mg). ^{1}H NMR (400 MHz, THF-d_8), δ (TMS, ppm): 8.00 (d, J = 8.8 Hz, 4H), 7.86 (s, 2H), 7.27 (td, J = 7.6, 0.8 Hz, 8H), 7.19–6.93 (m, 62H). ^{13}C NMR (100 MHz, THF-d_8), δ (TMS, ppm): 155.13, 148.86, 148.58, 146.81, 145.03, 144.74, 144.61, 144.56, 143.10, 141.71, 141.69, 139.29, 133.16, 132.77, 132.21, 132.17, 131.97, 130.81, 130.19, 128.47, 128.41, 128.38, 128.01, 127.22, 127.10, 126.95, 126.01, 124.74, 123.78, 123.40. HRMS (MALDI-TOF, *m*/*z*): $[M^+]$ calcd for $C_{106}H_{76}N_6S$, 1465. 5886; found, 1465.5864.

Preparation of TTA: The compound was prepared from compound **10**, (134 mg, 0.3 mmol), *N^1,N^1,N^4-triphenylbenzene-1,4-diamine* **9**, (222 mg, 0.66 mmol), $Pd(OAc)_2$ (22.4 mg, 0.1 mmol), P(*t*-Bu)$_3$·HBF_4 (87 mg, 0.3 mmol) and Cs_2CO_3 (780 mg, 2.4 mmol), following the same procedure described above. Orange red solid; yield: 59.2% (170 mg). ^{1}H NMR (400 MHz, THF-d_8), δ (TMS, ppm): 8.01 (d, J = 8.8 Hz, 4H), 7.87 (s, 2H), 7.32–7.19 (m, 20H), 7.11–7.09 (m, 12H), 7.06–7.03 (m, 6H), 7.01–6.97 (m, 4H). HRMS (MALDI-TOF, *m/z*): [M^+] calcd for $C_{66}H_{48}N_6S$, 956. 3661; found, 956.3667.

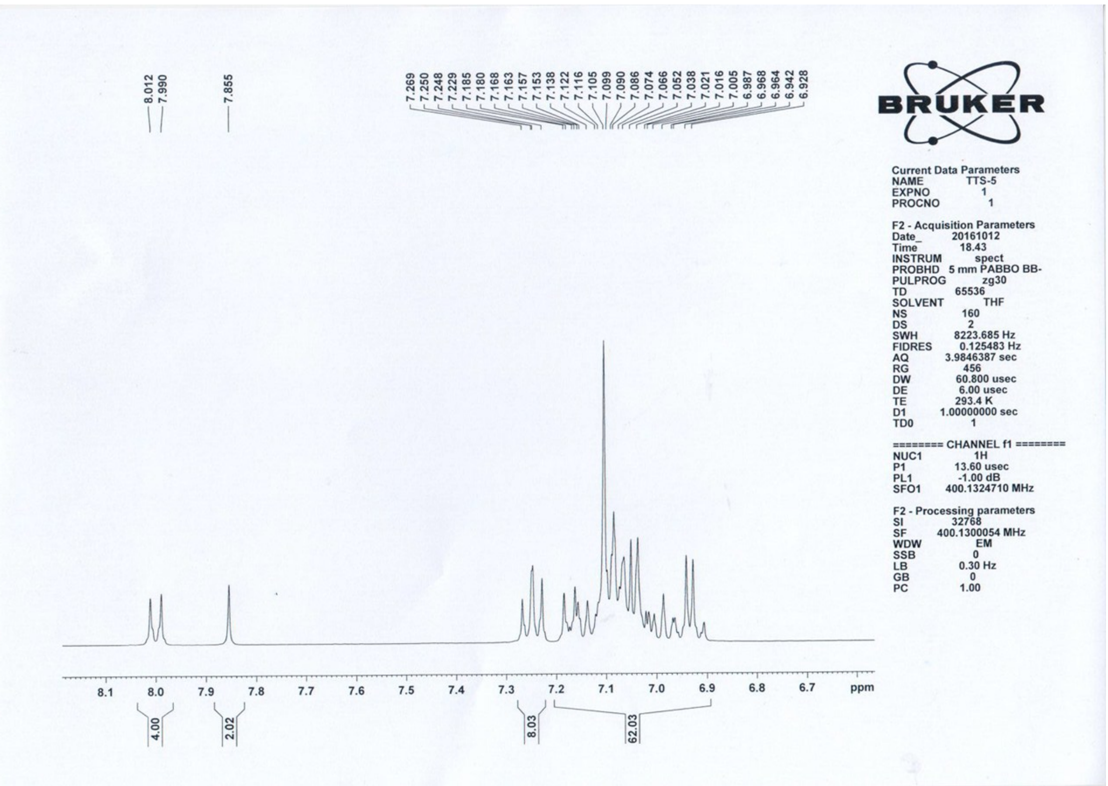

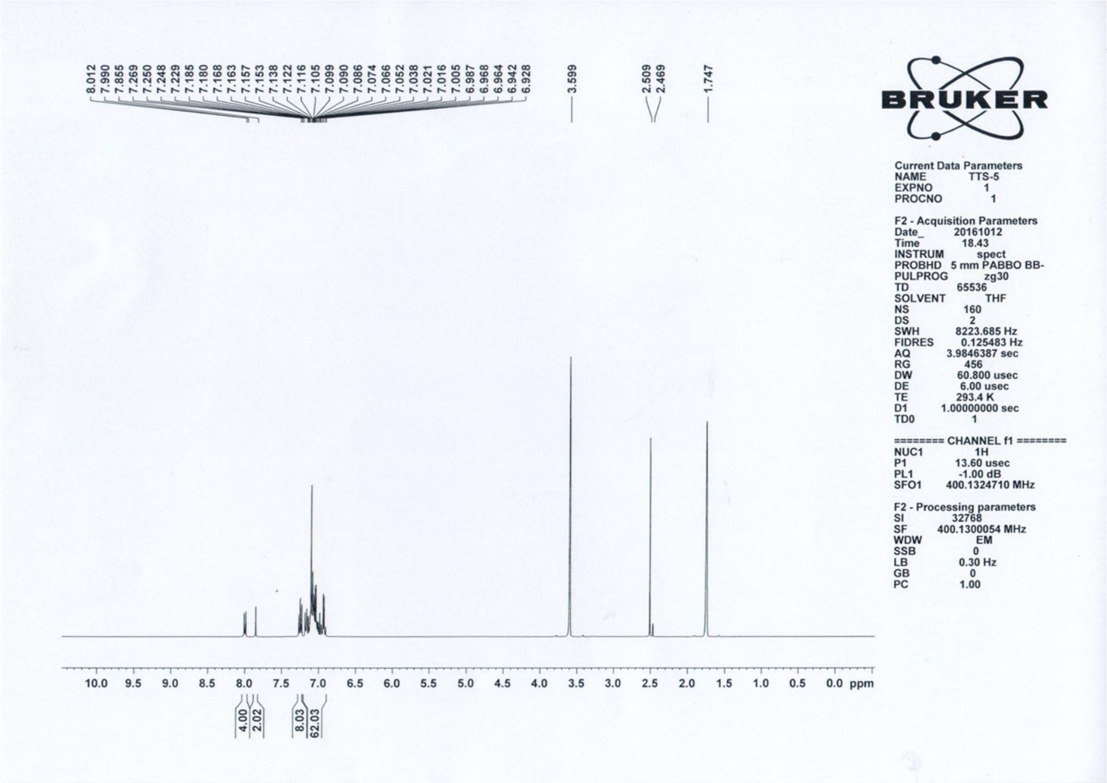

Fig. S1 ^{1}H NMR spectra of TTS in THF-d_8.

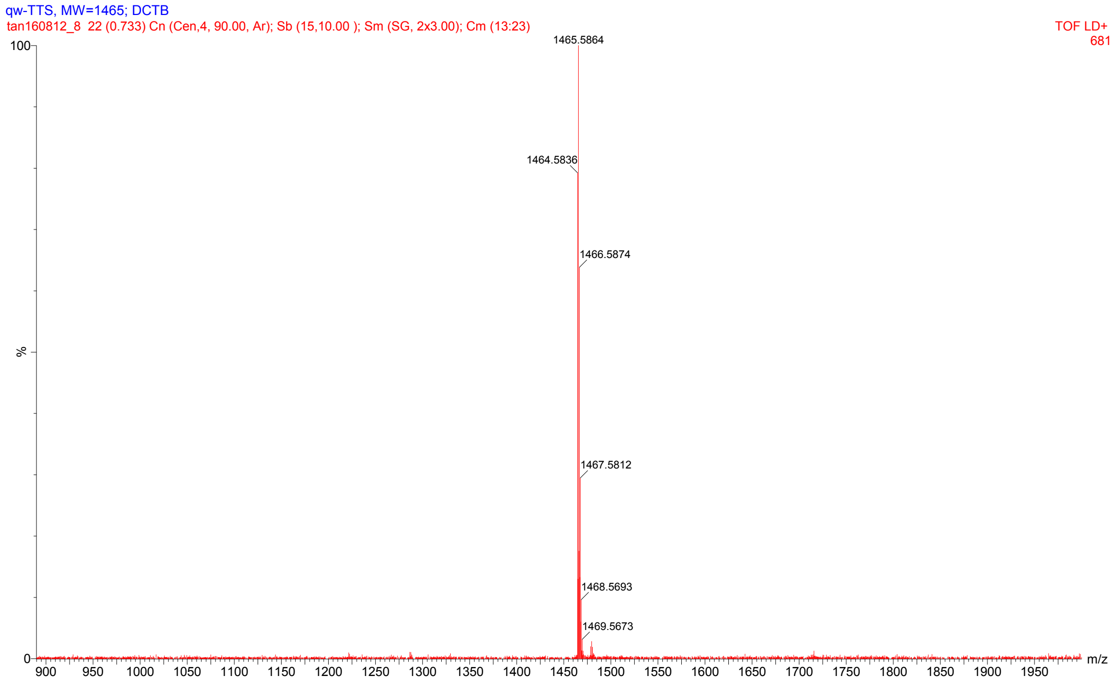

Fig. S2 HRMS spectrum of TTS.

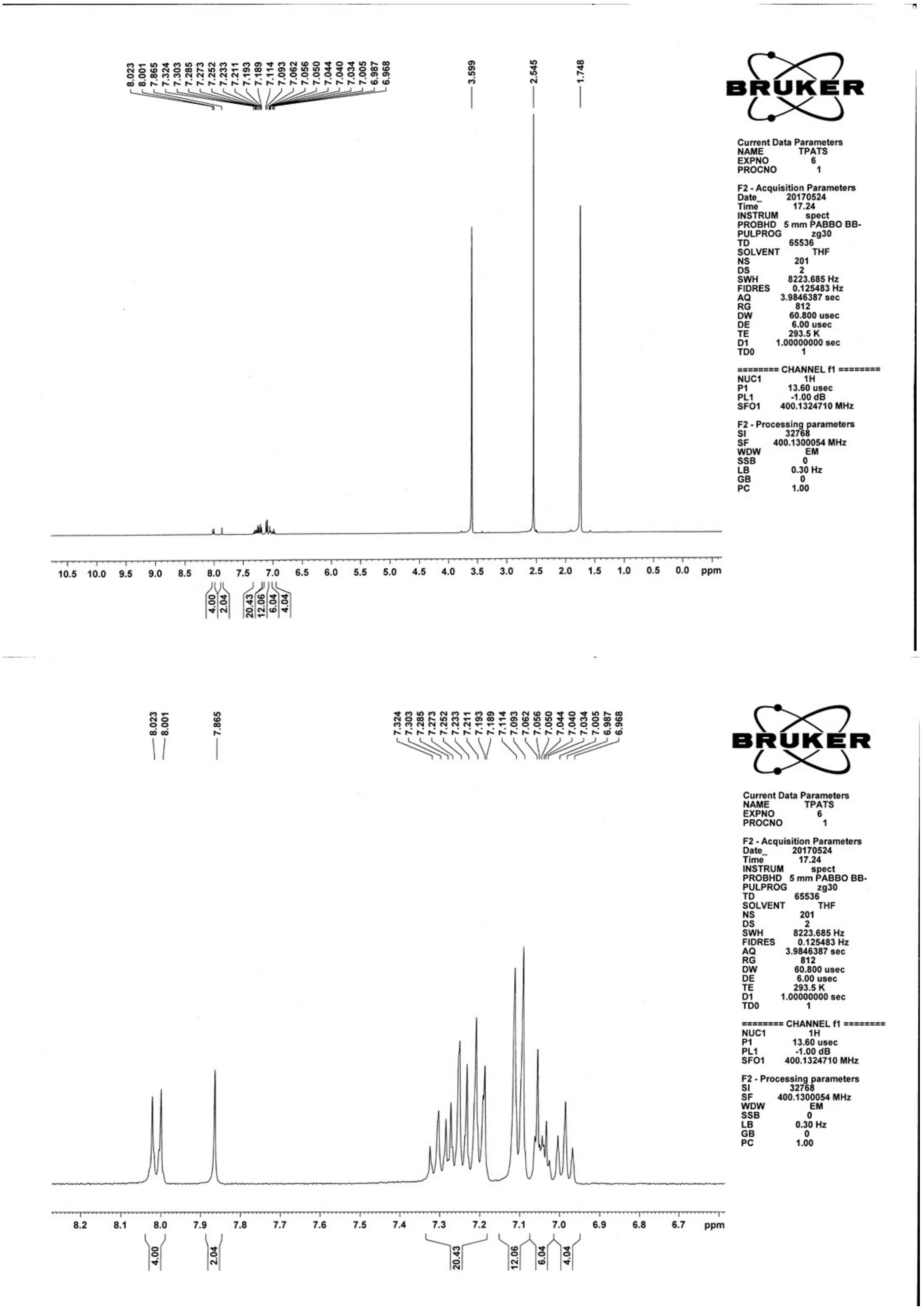

Fig. S3 ^{1}H NMR spectra of TTA in THF-d_8.

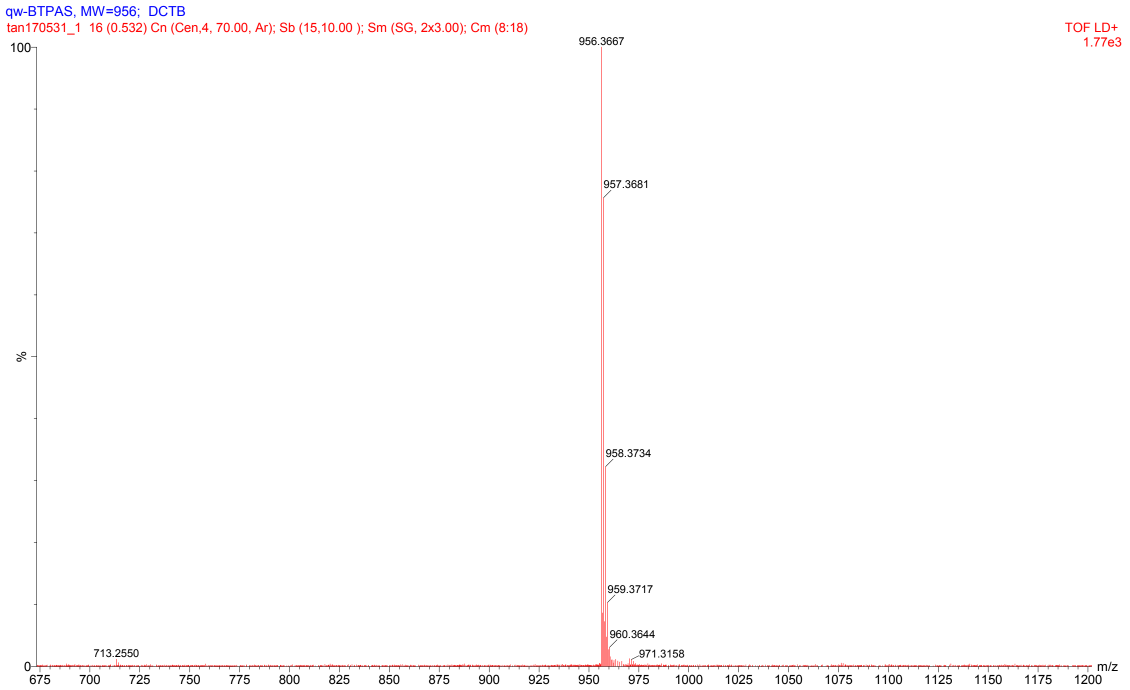

Fig. S4 HRMS spectrum of TTA.

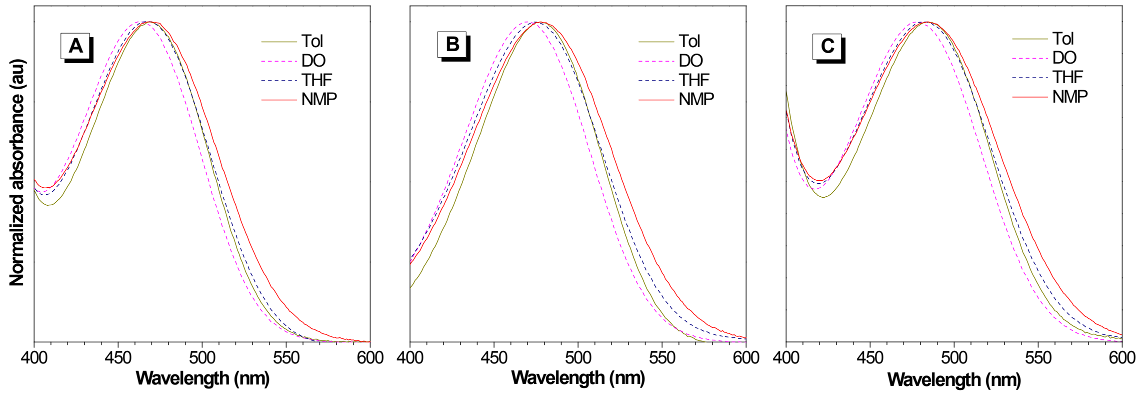

Fig. S5 Normalized absorption spectra of (A) TTB, (B) TTA and (C) TTS in solvents with different polarities.

Table S1 Absorption and emission of TTB, TTA and TTS in different solvents.

	TTB		TTA		TTS	
Solvent	λ_{ab} (nm)[a]	λ_{em} (nm)[b]	λ_{ab} (nm)[a]	λ_{em} (nm)[b]	λ_{ab} (nm)[a]	λ_{em} (nm)[b]
Tol	470	596	478	620	484	623
DO	465	609	471	632	478	640
THF	469	618	474	nd[c]	481	nd[c]
NMP	469	654	478	nd[c]	484	nd[c]

[a] λ_{ab} = absorption maximum. [b] λ_{em} = emission maximum. [c] nd = not detected.

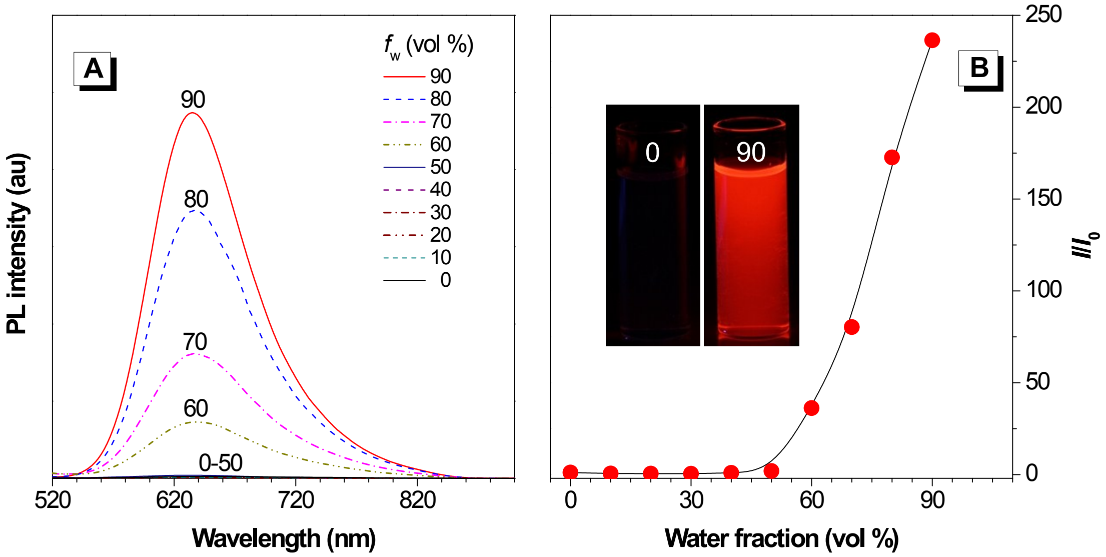

Fig. S6 (A) PL spectra of TTA in THF/water mixtures with different water fractions (f_w). (B) Plot of relative PL intensity (I/I_0) versus the composition of THF/water mixture of TTA. I_0 = emission intensity in pure THF solution. Concentration: 10 μM; excitation wavelength: 480 nm. Inset: fluorescent photographs of TTA in THF (f_w = 0%) and THF/water mixture with f_w = 90% taken under 365 nm UV illumination.

Table S2 Optical properties of TTS.

	λ_{ab} (nm)^a	λ_{em} (nm)^b			τ (ns)^d	E_g (eV)^e	HOMO/LUMO(eV)^f
		soln (Φ_F)^c	aggr	powder (Φ_F)^c			
TTS	481	nd (0.35)	636	636 (34.1)	1.86	2.21 (2.29)	−5.05 (−4.52)/ −2.84 (−2.23)

^{a} λ_{ab} = absorption maximum in THF. ^{b} λ_{em} = emission maximum in THF solution (soln), THF/water mixture (1:9 by volume) (aggr). ^{c} Fluorescence quantum yield (Φ_F,%) of THF solution and solid powders given in the parentheses. ^{d} fluorescence lifetime of solid powders (τ). ^{e} E_g = energy band gap calculated from the onset of the absorption spectrum. ^{f} HOMO = highest occupied molecular orbitals calculated from the onset oxidation potential, LUMO = lowest unoccupied molecular orbitals estimated by using the equation: LUMO = HOMO + Eg. The values in the parentheses are derived from theoretical DFT calculations. nd = not detected.

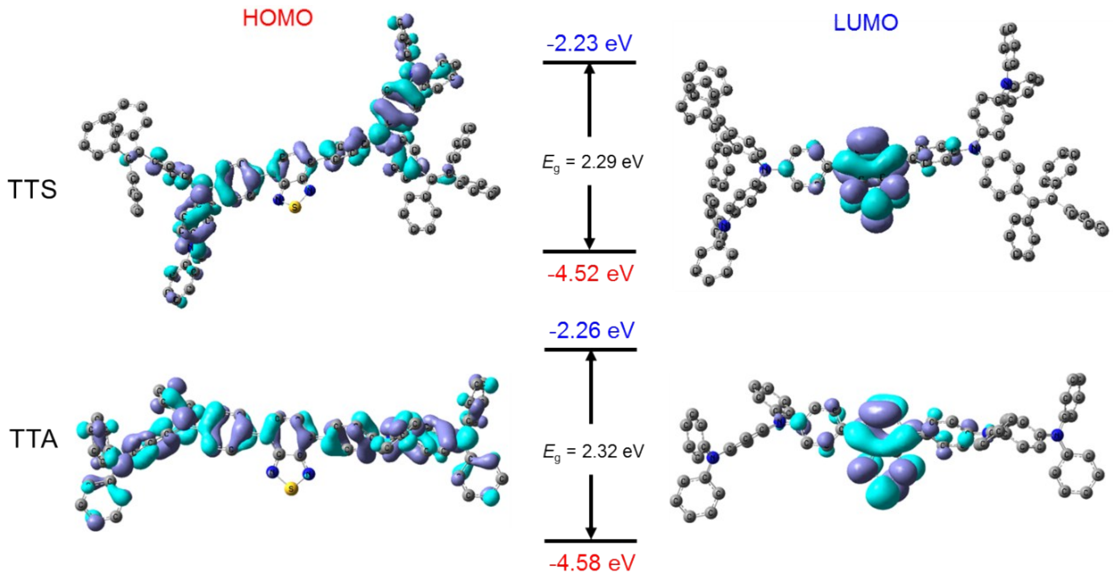

Fig. S7 Optimized geometries and molecular orbital amplitude plots of HOMO and LUMO levels of TTS and TTA calculated using the B3LYP/6-311G(d) basis set.

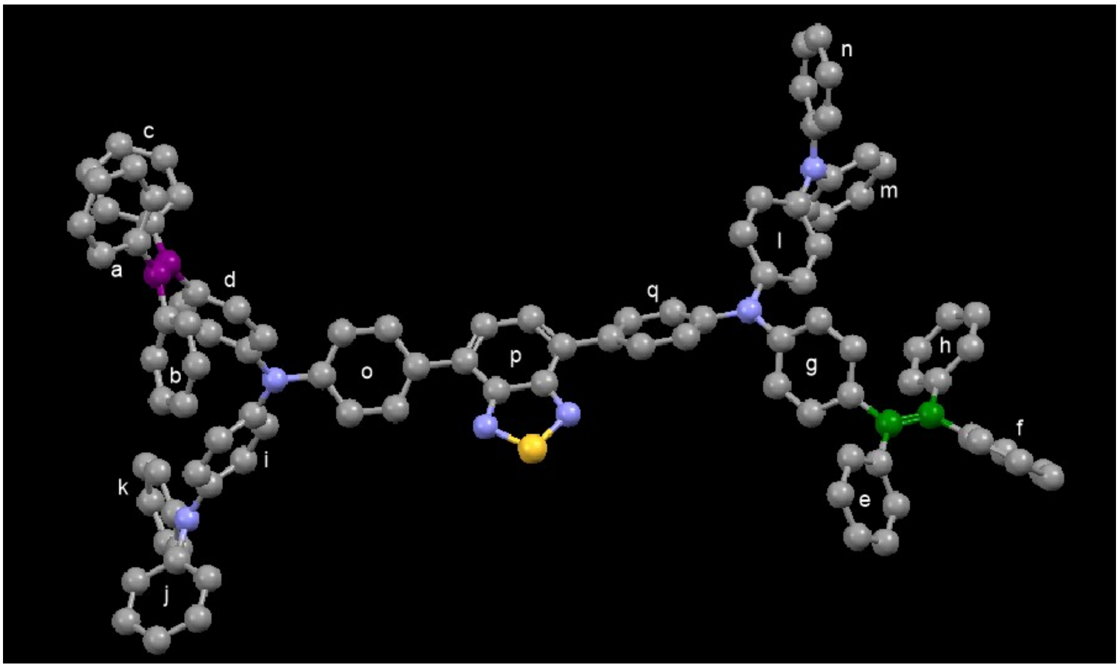

Fig. S8 Molecular geometries of TTS optimized at the DFT level of theory using the B3LYP/6-31G(d) basis set with Grimme's Empirical Dispersion correction (GD3).

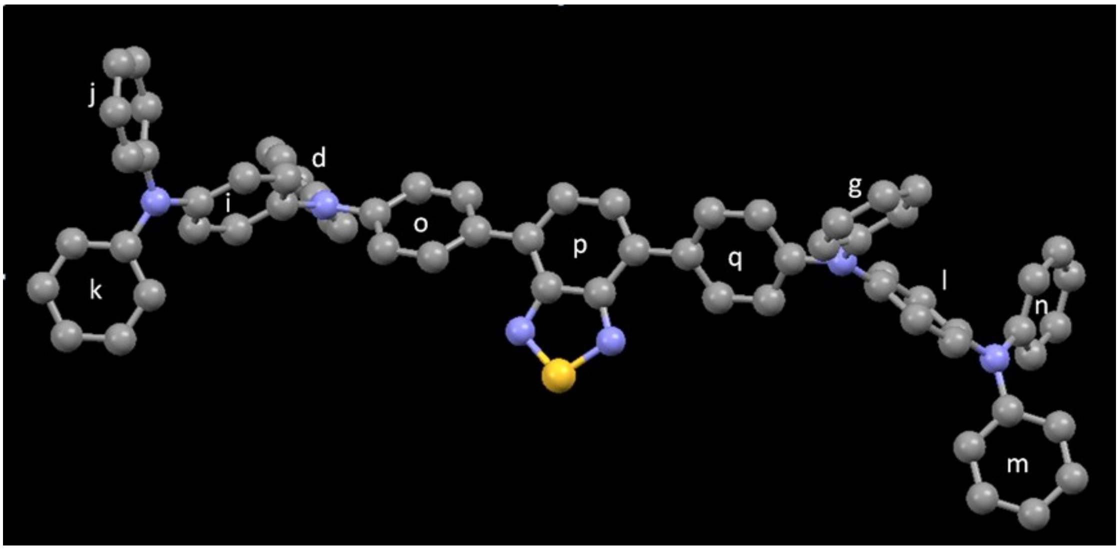

Fig. S9 Molecular geometries of TTA optimized at the DFT level of theory using the B3LYP/6-31G(d) basis set with Grimme's Empirical Dispersion correction (GD3).

Table S3 Summary of angles between different aryl rings or double bonds of TTS.

Rings and rings[a]	Torsion angles (°)	Rings and bonds[b]	Torsion angles (°)
d-o	68.02	a-D_p	116.52
i-o	67.08	b-D_p	115.24
o-p	36.56	c-D_p	135.31
p-q	143.30	d-D_p	132.24
g-q	109.27	e-D_g	120.93
l-q	116.33	f-D_g	136.28
i-j	106.24	g-D_g	107.38
i-k	73.60	h-D_g	131.09
j-k	108.03		
l-m	72.81		
l-n	73.35		
m-n	71.66		

[a] a, b, c, d, e, f, g, h, i, j, k, l, m, and n are different aryl rings as labeled in Fig. S8, d-o, i-o, o-p, p-q, g-q, and l-q mean the torsion angles between different aryl rings. [b] D_p and Dg are the double bonds which are highlighted in purple and green as shown in Fig. S8 a-D_p, b-D_p, c-D_p, d-D_p, e-D_g, f-D_g, g-D_g and h-D_g imply the torsion angles between different aryl rings and the near double bond which are highlighted in purple or green.

Table S4 Summary of angles between different aryl rings of TTA.

Rings and rings[a]	Torsion angles (°)	Rings and bonds[a]	Torsion angles (°)
o-p	36.14	m-n	112.03
o-d	113.56	g-l	70.34
o-i	112.29		
i-j	111.52		
i-k	68.56		
j-k	112.03		
i-d	70.34		
p-q	36.14		
g-q	66.45		
l-q	67.71		
l-m	111.44		
l-n	68.48		

[a] j, k, i, d, o, p, q, g, l, m, and n are different aryl rings as labelled in Fig. S9, o-p, o-d, o-i, i-j, i-k, j-k, i-d, p-q, g-q, l-q, l-m, l-n, m-n and g-l mean the torsion angles between different aryl rings.

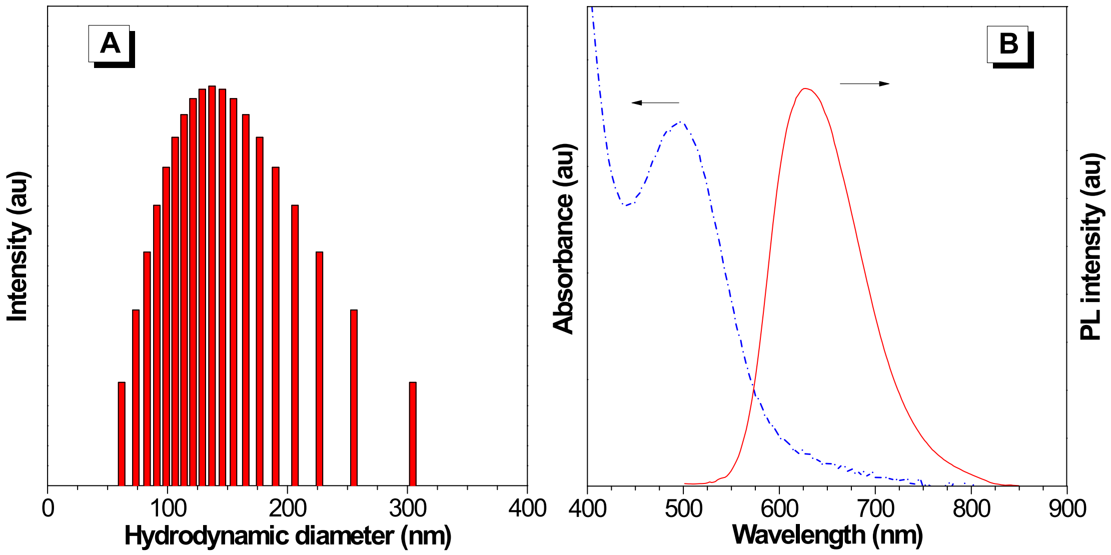

Fig. S10 (A) Particle size distribution and (B) absorption and emission spectra of TTS dots suspended in water. Excitation wavelength = 488 nm.

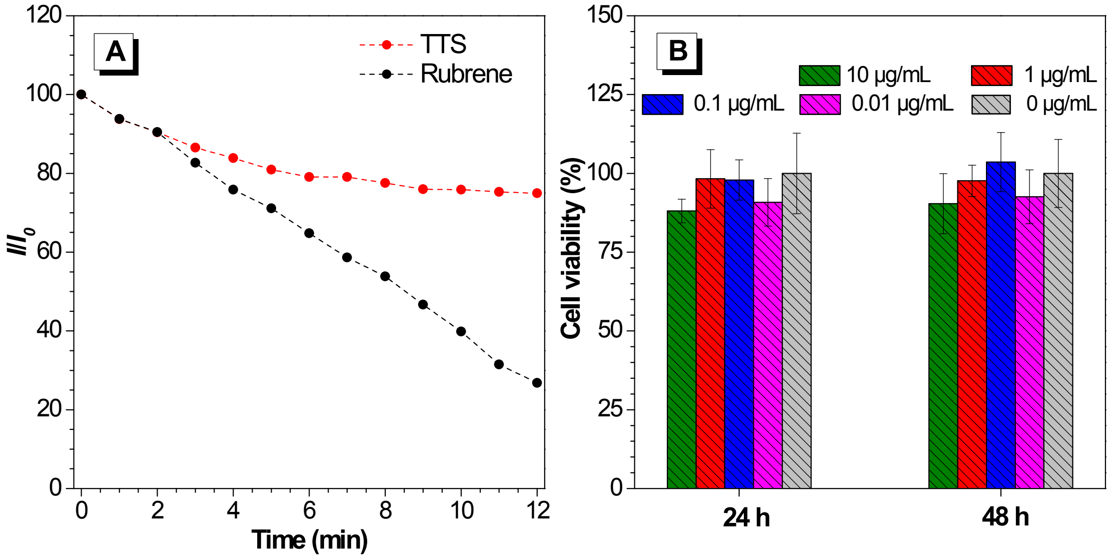

Fig. S11 (A) Photostability of TTS dots under continuous scanning at 480 nm using 450W Xe lamp. I_0 is the initial PL intensity, while I is the intensity after irradiation for a designated time interval. (B) Cell viability of A549 cells after incubation with 0, 0.01, 0.1, 1, and 10 μg/mL TTS dots for 24 h and 48 h.

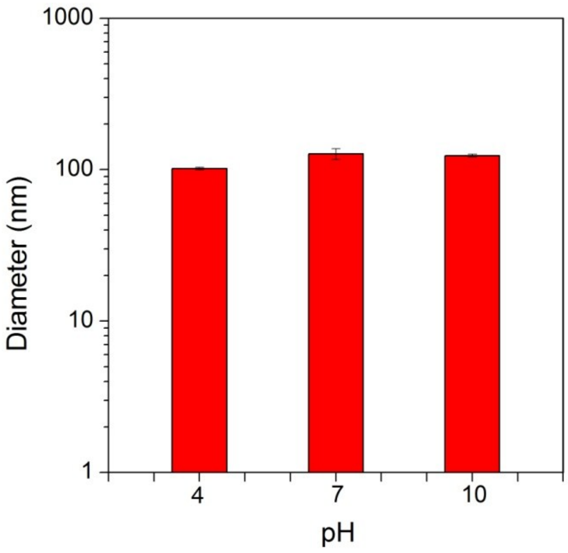

Fig. S12 Hydrodynamic diameters of the TTS dots in buffer solution with different pH (4.0, 7.0, and 10.0).

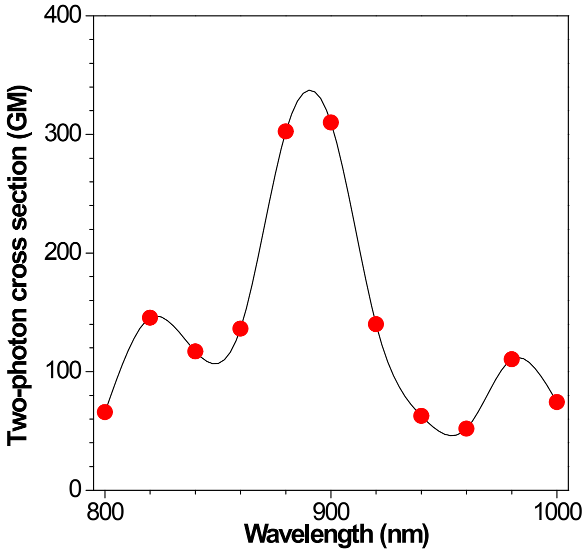

Fig. S13 Two-photon cross section of TTS in THF/water suspension (*with 99% water fraction*) with a concentration of 10 μM at different excitation wavelengths. (1GM = 1×10^{-50} cm^4 s $photo^{-1}$ $molecule^{-1}$)

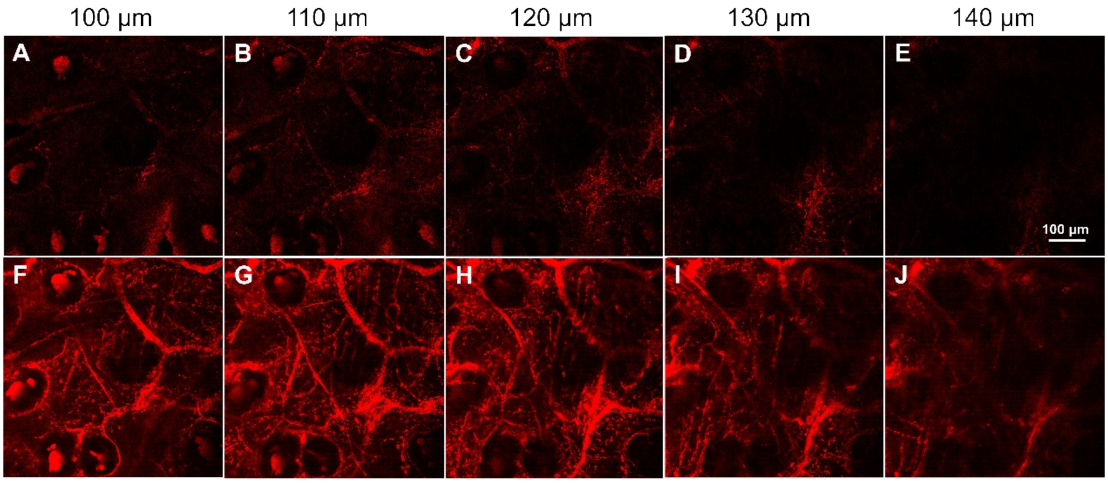

Fig. S14 (A–E) One-photon confocal luminescence and (F–J) two-photon scanning luminescence images of the blood vessels of mouse ear intravenously injected with TTS dots at penetration depth of 100–140 μm. Scale bar: 100 μm.

Notes and references

1. D. Lumpi, B. Holzer, J. Bintinger, E. Horkel, S. Waid, H. D. Wanzenbock, M. Marchetti-Deschmann, C. Hametner, E. Bertagnolli, I. Kymissis, J. Frohlich, *New J. Chem.* 2015, **39**, 1840–1851.

2. X. F. Duan, J. Zeng, J. W. Lü, Z. B. Zhang, *J. Org. Chem.* 2006, **71**, 9873–9876.

3. (*a*) W. Qin, K. Li, G. Feng, M. Li, Z. Yang, B. Liu, B. Z. Tang, *Adv. Funct. Mater.* 2014, **24**, 635–643; (*b*) J. Liu, L. Bu, J. Dong, Q. Zhou, Y. Geng, D. Ma, L. Wang, X. Jing, F. Wang, *J. Mater. Chem.* 2007, **17**, 2832–2838.
